# Sequential patterning of dynamic brain states distinguish Parkinson’s disease patients with mild cognitive impairments

**DOI:** 10.1016/j.nicl.2025.103779

**Published:** 2025-04-14

**Authors:** Aaron S. Kemp, A. Journey Eubank, Yahya Younus, James E. Galvin, Fred W. Prior, Linda J. Larson-Prior

**Affiliations:** a Department of Biomedical Informatics, 4301 W. Markham St., Little Rock, AR 72205, United States; b Department of Neurology, 4301 W. Markham St., Little Rock, AR 72205, United States; c Department of Neurobiology & Developmental Sciences, 4301 W. Markham St., Little Rock, AR 72205, United States; d Department of Pediatrics, at the University of Arkansas for Medical Sciences (UAMS), 4301 W. Markham St., Little Rock, AR 72205, United States; e Arkansas Children’s Research Institute, 13 Children’s Way, Little Rock, AR 72202, United States; f Little Rock Central High School, 1500 S. Little Rock Nine Way, Little Rock, AR 72202, United States; g Department of Neurology, University of Miami, Miller School of Medicine, Comprehensive Center for Brain Health, 7700 W Camino Real, Suite 200, Boca Raton, FL 33433, United States

**Keywords:** Brain states, Dynamic functional network connectivity (dFNC), Functional magnetic resonance imaging (fMRI), Machine learning, Mild cognitive impairment (MCI), Parkinson’s disease, Sequential pattern mining

## Abstract

Parkinson’s disease (PD) is a neurodegenerative disease which presents clinically with progressive impairments in motoric and cognitive functioning. Pathophysiologic mechanisms underlying these impairments are believed to be attributable to a breakdown in the spatiotemporal coordination of functional neural networks across multiple cortical and subcortical regions. The current investigation used resting state, functional magnetic resonance imaging (rs-fMRI) to determine whether the temporal characteristics or sequential patterning of dynamic functional network connectivity (dFNC) states could accurately distinguish among people with PD who had normal cognition (PD-NC, n = 18), those with PD who had mild cognitive impairment (PD-MCI, n = 15), and older-aged healthy control (HC, n = 22) individuals. Results indicated that the proportion of time during the rs-fMRI scan that was spent in each of three identified dFNC states (dwell time) differed among these three groups. Individuals in the PD-MCI group spent significantly more time in a dFNC state characterized by low functional network connectivity, relative to participants in both the PD-NC (p = 0.0226) and HC (p = 0.0027) cohorts and tend to spend less time in a state characterized by anti-correlated thalamo-cortical connectivity, relative to both the PD-NC (p = 0.016) and HC (p = 0.0562) groups. A machine-learning method using sequential pattern mining was also found to distinguish among the groups with moderate accuracies ranging from 0.53 to 0.80, revealing distinct sequential patterns in the temporal ordering of dFNC states. These findings underscore the potential of dFNC and sequential pattern mining as relevant methods for further exploration of the pathophysiologic underpinnings of cognitive impairment among people living with PD.

## Highlights

• Pathophysiology of cognitive impairment in Parkinson’s disease is poorly understood.
• Brain states were derived from dynamic functional network connectivity during rest.
• Brain state sequences were mined for patterns and compared using machine learning.
• Patterns distinguished Parkinson’s disease patients with/without cognitive deficits.
• Sequential patterning of brain states offers novel features for future investigations.

## 1 Introduction

Parkinson’s disease (PD) is a neurodegenerative disease initially presenting with motor symptoms, but also frequently manifests cognitive symptomatology. While research into the pathophysiologic mechanisms underlying the expression of motor abnormalities among patients with PD has implicated a likely causal connection to the loss of dopaminergic projections from the striatum, the neurologic mechanisms associated with cognitive impairment in patients with PD are not as well understood (Bohnen and Albin, 2011). These cognitive impairments manifest clinically across a range of discernable phases, from cognitively normal PD (PD-NC), to mild cognitive impairment due to PD (PD-MCI), and eventual progression to PD with dementia (Litvan et al., 2011, Postuma et al., 2015, Saredakis et al., 2019). While the clinical presentation and time course are quite variable across individuals with PD (Jellinger, 2024), executive, attentional, and visuoperceptual function deficits generally characterize early cognitive decline (Johnson et al., 2016, Kalbe et al., 2016, Yilmaz et al., 2016) and can include impairments in attentional set-shifting, working memory, sequencing of complex actions, misperceptions, and behavioral regulation (Fallon et al., 2016, Machado et al., 2016, Muslimovic et al., 2005, Stuart et al., 2016). Although, dopaminergic dysfunction is largely suspected, cholinergic signaling has also been shown to play a role in the expression of attention and cognitive flexibility, with cholinergic deficits in striatum (Perez-Lloret and Barrantes, 2016) and midbrain cholinergic centers (Schulz et al., 2018) showing associations with reduced ability to adaptively switch between behavioral sequences (Gut and Mena-Segovia, 2019). These findings point to the impact of broad cortico-subcortical functional networks on motor and non-motor dysfunctions in PD, including the progressive decline in cognition. Ideally, a method that could capture the dynamic engagement or the relative temporal disruption of coordinated activity across these functional neural networks could help elucidate the suspected mechanisms underlying the progression of cognitive impairments among people living with PD.

Functional magnetic resonance imaging (fMRI) can be used to characterize resting state networks (RSNs) by identifying areas which have a high degree of shared temporal variance in the blood oxygenation level dependent (BOLD) signal across cortical and subcortical regions (Fox and Raichle, 2007). Dynamic functional network connectivity (dFNC) has recently emerged as a method of characterizing sequential changes in the degree of functional coupling among RSNs across time within a single fMRI scan (Allen et al., 2014, Díez-Cirarda et al., 2018, Filippi et al., 2023, Lurie et al., 2020, Shine et al., 2016, Smith et al., 2013, Vidaurre et al., 2018). Although there are several different methods of calculating dFNC (Calhoun et al., 2014) one of the most common is obtained by the Group ICA for fMRI Toolbox (GIFT) (Calhoun et al., 2001, Rachakonda et al., 2010), which utilizes sliding time windows and k-means clustering to identify distinct “states” that have differing degrees of functional connectivity among RSNs.

Previous investigations have shown that the proportional amount of time spent in each of these time-varying dFNC states can reveal distinctive “chronnectome” characteristics that are predictive of functional cognitive abilities (Fan et al., 2020, Hilger et al., 2020, Liao et al., 2017, Liu et al., 2018, Ramirez-Mahaluf et al., 2020) and significant differences when compared across individuals with and without various brain disorders (Cao et al., 2023, Damaraju et al., 2014, Fiorenzato et al., 2019, Gan et al., 2021; 1764., Iraji et al., 2019, Li et al., 2021, Rashid et al., 2014, Reinen et al., 2018, Tan et al., 2023, Wang et al., 2016, Wu et al., 2024, Xin et al., 2024, Xu et al., 2022, Yi et al., 2022, Zhu et al., 2021). Several recent investigations have also demonstrated that the temporal ordering of these emergent dFNC states could yield important markers of abnormal neural network engagement. Such measures of temporal or sequential organization among transitions have been posited as pathologic markers of information processing deficits among individuals with certain brain disorders (Chen et al., 2023, Jie et al., 2018, Sun et al., 2019), while others have suggested that these measures may be attributable to treatment-related effects of therapeutic dopamine modulation (Ramirez-Mahaluf et al., 2023). Although, the current investigation was not designed to assess whether the temporal ordering of dFNC brain states is more likely to be related to disease progression or medication effects, it does offer the opportunity to explore whether the sequential patterning of these brain states can accurately distinguish among healthy control participants and those diagnosed with PD, with or without mild cognitive impairments.

Our analysis expands on the previous literature by combining GIFT to visualize group differences in RSNs with a novel machine-learning method of sequential pattern mining called Seq2Pat (Ghosh et al., 2022, Kadıoğlu et al., 2023, Wang et al., 2022). This innovative analysis investigates alterations in dynamic local and global connectivity, network reorganization, and defines distinct sequential patterns in the temporal ordering of states between HC, PD-NC, and PD-MCI. The aim of our data-driven study is to explore if we can detect dFNC alterations between PD-MCI from PD-NC in relation to HC to gain insight into PD’s impact on the temporal sequencing of dFNC state transitions. Our research is one of the first analyses to supplement dFNC methods with sequential pattern mining techniques.

## 2 Material and methods

### 2.1 Participant demographics

Forty-three participants clinically diagnosed with Parkinson’s disease (PD) and thirty-nine healthy control (HC) participants were recruited into a 2-year study of cognitive impairment in PD at New York University (NYU), Grossman School of Medicine. All 82 participants provided written informed consent before completing any study procedures, however only 62 participants (36 PD and 26 HC) partook in the optional neuroimaging scans described below. Among those with available imaging data, 4 participants from the HC group, 2 from the PD-NC group, and 1 from the PD-MCI group were excluded due to scanning and motion artifacts. Accordingly, a total of 55 participants (all right-handed) were included in the current investigation: 22 in the HC group, 18 in the PD-NC group, and 15 in the PD-MCI group. The data for all consenting participants was collected at the Center for Biomedical Imaging at NYU, where the data were de-identified and transferred to the University of Arkansas for Medical Sciences for further investigation.

Table 1 summarizes participant demographics, years since PD diagnoses, measures of disease severity, and general cognitive function assessment scores. Disease severity was assessed by the MDS-Unified Parkinson Disease Rating Scale (MDS-UPDRS) (Goetz et al., 2008) and the Hoehn & Yahr Scale (Hoehn and Yahr, 1967). Measures of cognitive performance included the Montreal Cognitive Assessment (MoCA) (Nasreddine et al., 2005) and the Repeatable Battery for the Assessment of Neuropsychological Status (RBANS) (Randolph et al., 1998). MoCA total scores less than 26 and scaled scores less than 85 on two or more cognitive domain subscales from the RBANS were regarded as evidence of impairment. Global cognitive status was defined by the Clinical Dementia Rating (CDR) (Morris, 1993), following a semi-structured interview with the participant and study partner, with CDR = 0 signifying the absence of dementia and CDR = 0.5 signifying very mild impairments. Classification of participants into their respective cohorts was determined by the NYU clinical team following consensus conference using neurological exam, clinical ratings scales, CDR, and neuropsychological testing as follows: (a) CDR = 0 and no parkinsonism was designated as healthy controls, (b) CDR = 0 and parkinsonism but no evidence of impairment present was designated as PD-NC, and (c) CDR = 0.5 with parkinsonism and evidence of impairments present was designated as PD-MCI. No participants were diagnosed with dementia. Statistical significance comparisons were calculated using a two-tailed *t*-test of the homoscedastic variables. Participants were assessed in the ‘ON’ stage during analysis, indicating they were taking their prescribed doses of dopaminergic medication during the study, however, exact dosing information is not available so it was not possible to calculate the levodopa-equivalent dosing for use as a co-variate.

**Table 1:** Demographics, PD Duration, Measures of PD Severity (MDS-UPDRS, Hoehn & Yahr), and General Cognitive Function Assessment Scores (MoCA, RBANS).

	Healthy	PD-NC	PD-MCI	Significant Differences
N(Sex: M/F)	22(7/15)	18(11/7)	15(11/4)	%M: HC < PD-NC, PD-MCI ^ⱡ^
Mean Age ± SD	70.7 ± 7.5	65.9 ± 5.2	69.9 ± 9.3	PD-NC < HC ^ⱡ^
Years of Edu. ± SD	16.3 ± 1.6	17.8 ± 2.3	18.1 ± 1.5	HC < PD-NC, PD-MCI ^ⱡ^
PD Duration ± SD	na	7.1 ± 4.4	7.5 ± 5.9	(not significant)
MDS-UPDRS ± SD	na	24.8 ± 10.9	29.7 ± 9.5	(not significant)
Hoehn & Yahr ± SD	na	2.4 + 0.6	2.4 + 0.6	(not significant)
MoCA ± SD	26.0 ± 2.3	27.1 ± 1.9	22.7 ± 3.5	PD-MCI < HC, PD-NCI ^ⱡ^
RBANS ± SD	107.9 ± 10.1	101.6 ± 16.1	82.8 ± 6.8	PD-MCI < HC, PD-NC *

ⱡ p < 0.05; * p < 0.0001; SD = standard deviation; na = not applicable.

### 2.2 Neuroimaging data acquisition and preprocessing

The MRI data were collected by the Center for Biomedical Imaging at NYU on a Siemens 3T Trio MRI scanner. The sessions were comprised of a 7-minute resting state fMRI (rs-fMRI) scan, structural (T1 and T2 weighted) anatomical scans, diffusion tensor imaging (not reported herein), and a B0 field map. The participants were instructed to lie in the scanner with eyes closed and to indulge in casual mind wandering but not to fall asleep. The rs-fMRI data were preprocessed in FMRIB Software Library (FSL) (Jenkinson et al., 2012) version 6.0. Preprocessing included brain extraction (BET) (Smith, 2002), slice-time and motion correction, and registration to the Montreal Neurological Institute (MNI) standard space T1, 2 mm brain (MNI 152). FEAT preprocessing used BOLD 4D data at 196 volumes with repetition time (TR) set at 2 s and filter cutoff of 100 (Woolrich et al., 2004). The pre-statistics options FSL MCFLIRT and B0 unwarping were utilized, and the selected field map produced from BET set to the Effective EPI: 0.59, EPI TE: 29 and unwarp direction −y with a signal loss of 10. Independent Component Analyses for the Automated Removal of Motion Artifacts (ICA-AROMA) (Pruim et al., 2015) was also used to correct for any remaining head motion. Interleaved slice timing correction and the BET brain extraction option was selected and spatial smoothing FWHM set to 6 mm. Intensity normalization, High pass filter, and MELODIC independent component analysis (ICA) were also selected. We registered the scans by uploading the T1 as the main structural image with the default values selected in FSL. To map our anatomical images to standard brain space we used the openly available MNI152 T1 2 mm brain and 12 DOFs for linear FLIRT (Jenkinson and Smith, 2001) and 10 mm warp for nonlinear FNIRT.

### 2.3 Dynamic functional network connectivity (dFNC) analyses

**Fig. 1:**
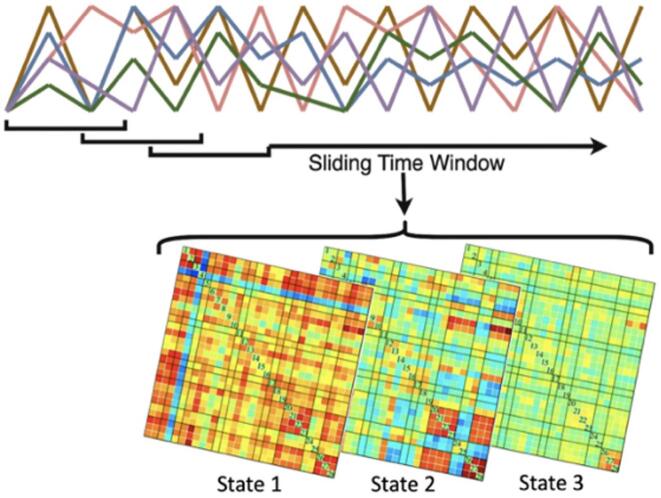
Displays the process of extracting dynamic functional network connectivity (dFNC) states from the time-courses through the sliding-time window and the k-means clustering methods.

The resulting denoised data were uploaded to the Group ICA fMRI Toolbox (GIFT; version 3.0b) to analyze dFNC and distinguish network differences between our 3 groups (Allen et al., 2011, Kim et al., 2017). In GIFT’s ICA user interface, the number of independent components (ICs) was set to 30 and the non-linear algorithm Infomax and ICASSO stability analysis were both selected. ICA was run 3 times on RandInit mode. Back reconstruction of the time courses and spatial maps were performed with group ICA, which conducts standard subject specific principal components analysis (PCA) through GIFT to reduce dimensionality of participant specific functional data. Group analysis mode was set to serial and all components were scaled using Z-scores. A total of 2 PCA data reductions were run based on the user specified settings outlined. The individual network component (IC) labels with a max correlation ≥0.20, as identified by GIFT’s internal component labeler, were analyzed for potential inclusion in the RSNs to ensure consistency and reliability of the identified networks. Using a combination of GIFT’s internal component labeler and visual inspection with a specialist (LLP), the validated ICs were separated into 11 RSN categories of biological interest: Auditory (AUD) network, Basal Ganglia (BG) network, Cerebellar (CB) network, Default Mode (DMN) network, Executive Control (EC) network, Frontal Parietal (FP) network, Language (LA) network, Salience (SAL) network, Sensorimotor (SM) network, Thalamus (TH) network, and Visual (VIS) network. Temporal dFNC was processed by repeating the L1 regularization method 10 times to calculate an inverse covariance matrix with a window size of 26 TR, a Gaussian Window Alpha Value of 3 and TR of 2. We used the elbow criterion of the cluster validity index to calculate the optimal number of states for our respective data set without over segregating the networks (Allen et al., 2014) and determined that 3 dFNC states (*k* = 3) explain the maximum amount of network variability resulting from the sliding-time window. Post-processing included the analysis of the time courses using the sliding-time window approach (Fig. 1) to calculate the co-variations in the functional connectivity across acquisition for each participant group, resulting in dFNC matrices (Allen et al., 2014, Díez-Cirarda et al., 2018, Kim et al., 2017). This method captures network activity at specified intervals during the scan allowing sampling at sequential time points of the intrinsic connectivity that occurs during resting state. Previous evidence argues that by using the sliding-time window technique, the mean dwell time spent in distinct brain states can distinguish levels of severity in PD (Díez-Cirarda et al., 2018, Kim et al., 2017). No covariates were regressed from the dFNC correlations, the *k*-means clusters were set to 3 and the ICA Infomax clustering algorithm was repeated 5 times using the City distance method.

### 2.4 Dichotomic pattern mining (DPM) using Seq2Pat

Following dFNC analyses using GIFT, the full sequence of dominant states across 170, 1-TR advancements of the 26-TR sliding window was output for each individual participant. These sequences were then used as the input for sequential pattern mining using Seq2Pat (Ghosh et al., 2022, Kadıoğlu et al., 2023, Wang et al., 2022). Seq2Pat is freely available as a Python library. It was originally developed to identify sequential patterns in click-stream digital behaviors among website users and employs a multi-valued decision diagram for Dichotomic Pattern Mining (DPM). The DPM step returns all sequential patterns identified across all subjects, in addition to those that are unique to each of the two groups in the specified dichotomy. In this step, the primary constraint is the “minimum support”, which is the minimum proportion of examples from either group that must contain at least one instance of a given pattern to be retained for further analyses. Seq2Pat then utilizes one-hot encoding to return vectors for all sequential patterns that are uniquely identified in one group relative to the other, at a given minimum support threshold. In the current analyses, the minimum support was set at 0.05, and the encoding vectors for all sequential patterns that were unique to either the HC participants or the participants living with PD were then separately used as the input features to train multiple, 2-way (HC vs PD, or PD-NC vs PD-MCI), machine-learning classification models using the “compare_models” function in PyCaret (Ali, 2020). For each model 80 % of the cases were used for training and 20 % were held out for testing. The hyperparameters for the best performing models [Random Forest (RF) and Linear Discriminant Analysis (LDA)] were then optimally tuned using the “tune-model” function in PyCaret, and the results of those were evaluated following 100 iterations of 10-fold cross-validation to identify the “most important” (i.e., most discriminative) features among the sequential patterns.

## 3 Results

From the original 62 consenting study participants for whom MRI data were available, severe movement-related or imaging artifacts precluded analysis of 4 participants from the HC group, 2 from the PD-NC group, and 1 from the PD-NC group. Therefore, all reported results are derived from analyses conducted on the remaining 55 participants, including 22 participants in the HC group, 18 in the PD-NC group, and 15 in the PD-MCI group. Statistical comparisons of the demographic information, cognitive performance scores, and clinical severity ratings for these 55 participants is summarized in Table 1, above.

### 3.1 Dynamic functional network connectivity (dFNC) analysis results

**Fig. 2:**
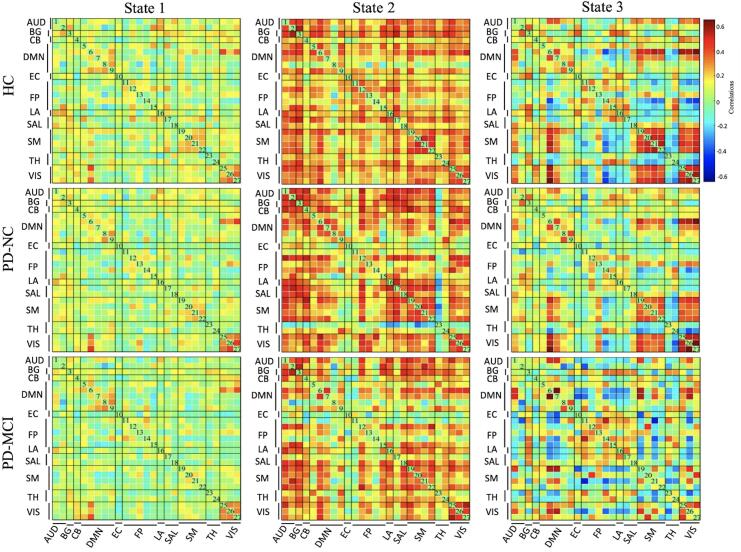
The dFNC matrices of each group separated by state. These networks explore the subtle network alterations between the participant groups across the scan. PD-MCI – Parkinson’s disease with mild cognitive impairment; PD-NC – Parkinson’s disease with normal cognition; HC – healthy controls; AUD – Auditory Network; BG – Basal Ganglia Network; CB – Cerebellar Network; DMN – Default Mode Network; EC – Executive Control Network; FP – Frontal Parietal Network; LA – Language Network; SAL – Salience Network; TH – Thalamus Network; VIS – Visual Network.

**Fig. 3:**
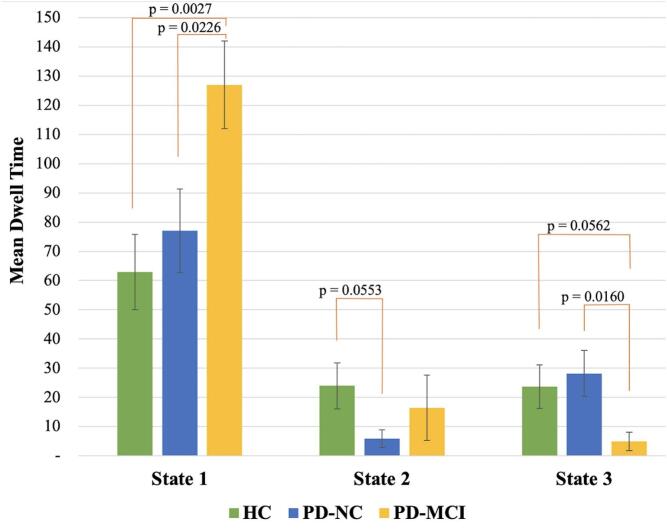
The mean dwell time spent in each state by group. Error bars indicate standard error of the means. PD-MCI – Parkinson’s disease with mild cognitive impairment; PD-NC – Parkinson’s disease with normal cognition; HC – healthy controls.

The dFNC analyses were conducted using the Group ICA fMRI Toolbox (GIFT), which returned 3 dynamic brain states, each defined by a unique configuration of functional connectivity among the 11 RSNs defined above. Fig. 2 shows the dFNC matrices indicating the degree of correlation (yellow to red) and anti-correlation (green to blue) between the RSNs of the 3 detected dFNC states, as averaged across instances of each state within each of the study groups. State 1 in Fig. 2 can be characterized as a hypo-connected state, based on the relatively sparse inter- and intra-network connectivity observed. State 2 in Fig. 2 can be characterized as a hyper-connected state, as there is a relatively high level of inter- and intra-network FC across RSNs. State 3 in Fig. 2 has a distinct pattern of correlated and anti-correlated functional connectivity among RSNs as seen in both the HC and PD-NC cohorts; however, this pattern is not clearly distinguishable among the participants in the PD-MCI group. As the PD-MCI group included the lowest number of participants and the lowest proportion of time spent in this state (as shown in Fig. 3, below), the differences in functional connectivity among RSNs in State 3 should be interpreted with caution, as these apparent differences could be attributable to a smaller number of instances of this state across individuals in the PD-MCI group.

The mean dwell time each respective cohort spent in the 3 dFNC states are presented in Fig. 3. Participants in the PD-MCI group spent significantly more time in the hypo-connected State 1 than did participants in the HC (p = 0.0027, t = −3.23) and PD-NC (p = 0.0226, t = −2.39) groups. Participants in the PD-MCI group also showed a marginally significant trend toward less time spent in State 3 relative to the HC group (p = 0.0562, t = 1.98) and significantly less time than the PD-NC (p = 0.0160, t = 2.55) group. For State 2, the participants in the PD-NC group trended toward spending less time in this hyper-connected state relative to those in the HC group (p = 0.0553, t = 1.98), but no significant differences were found with the PD-MCI group for this state.

### 3.2 Dichotomic pattern mining (DPM) results

**Fig. 4:**
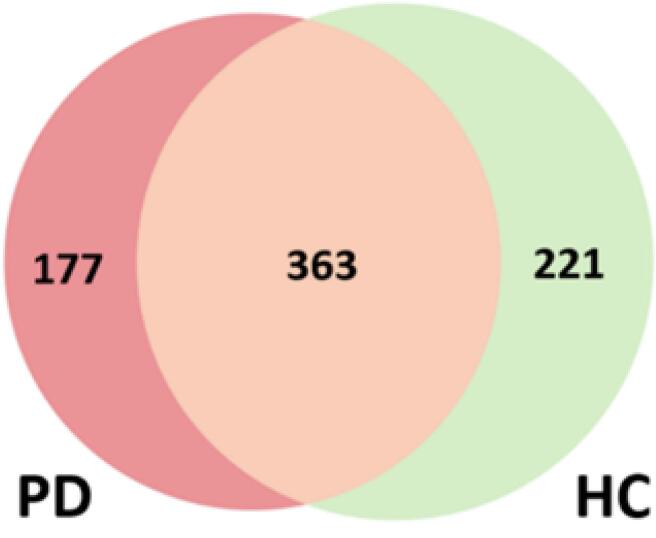
Venn diagram comparing the number of distinct patterns the patients with Parkinson’s disease (PD) and healthy controls (HC) displayed and the number of patterns they had in common across the scanning period.

As depicted in Fig. 4, the DPM method from the Seq2Pat Python library (Ghosh et al., 2022, Kadıoğlu et al., 2023, Wang et al., 2022), using a minimum support of 0.05 and no time constraints, yielded a total of 761 sequential patterns. Of those, 177 were unique to participants with PD, 221 were unique to the HC individuals, and the remaining 363 were found across individuals in either group. One-hot encoding was then utilized to note the presence of the patterns identified by the DPM across individual participants’ entire sequence of state transitions. These encoding vectors were then used as input for a comparison of machine-learning, classification models using the PyCaret Python library (Ali, 2020). Both the PD Sequential Patterns (shown in red) and the HC Sequential Patterns (shown in green) were separately input to train the classification models, and an additional model was trained on the Percent Dwell Times (white background in Table 2). The models that were consistently found to yield the best results were the Random Forest (RF) and Linear Discriminant Analysis (LDA) models.

**Table 2:** Performance of RF or LDA classification models trained on state dwell times (white background) or sequential patterns found in  or  groups following 100 iterative fits using 10-fold cross validation.

A summary of the performance of these models is provided in Table 2, including the mean values for accuracy, area under the receiver operating characteristic curve (AUC), recall, precision, and F1 scores for the tuned models, following 100 iterative fits using 10-fold cross validation. Note that Table 2 includes separate results for the models that were trained on the dwell time percentages for each state (white background), models that were trained on the patterns that were detected among the HC individuals (green background), and the models that were trained on the patterns that were detected among the participants with PD (red background). Furthermore, Table 2 also includes the results of classification models that were trained to distinguish between the subgroups of PD participants who either had normal cognition (PD-NC) or manifested mild cognitive impairments (PD-MCI), using either the HC patterns (green) or the PD patterns (red) as the input features. For that classification task, note that the models trained on the PD patterns performed considerably better than the models trained on the HC patterns, while the reverse was true for the classification of HC versus PD.

Table 3 provides a listing of the sequential patterns that were identified as being the “most important” (i.e., most discriminative) features by the best performing models from Table 2. These sequential patterns depict the dominant state for each 1-TR advancement of the 26-TR sliding window. For the classification of HC versus all PD individuals, when trained on patterns that were found among the HC participants (green), the most discriminative features were predominantly patterns that transition from State 2 to State 1 and then back to State 2 or State 3. When trained on patterns that were unique to the PD group (red), the most discriminative features were mostly those that transition from State 1 to State 2 or State 3 and back to State 1. For the classification of PD-NC versus PD-MCI, the most discriminative features from the PD cohort (red) were those that started in State 1 briefly transitioned to State 3 then back to State 1, but also included several that transitioned from State 1 to State 3 and then to State 2.

**Table 3:** Top 10 “most important” sequential patterns for classifying either HC versus PD, or PD-NC versus PD-MCI, by model and input features used in training ( or  patterns).

## 4 Discussion

Our study investigated dynamic functional network connectivity (dFNC) alterations between Parkinson’s disease patients with mild cognitive impairment (PD-MCI), those with normal cognition (PD-NC), and healthy controls (HC) using resting-state fMRI, novel sequential pattern mining, and machine learning techniques. We found some significant and some marginally significant differences in the mean dwell times that these groups spend in the three dFNC states that we identified using GIFT. Machine-learning classifiers trained on these state dwell times were also found to be reasonably accurate in distinguishing HC individuals from participants living with PD, as well as in distinguishing the participants with PD-MCI from those with PD-NC. By comparison, the classifiers trained on the sequential patterns of state transitions that were found to be unique among HC participants (shown in green) performed slightly better at distinguishing between HC and PD, but worse at distinguishing PD-NC from PD-MCI, relative to the classifiers trained on state dwell times. However, the classifiers that were trained on the sequential patterns that were unique to the participants with PD (shown in red), performed better at distinguishing individuals with PD-NC from those with PD-MCI. This finding would appear to indicate that the presence of “abnormal” patterns of sequential state transitions may be more discriminative than the absence of “normal” patterns for the detection of cognitive impairments among people with PD.

Understanding the neural basis of manifest impairments in cognitive functioning requires modeling the dynamic transitions in functional network connectivity. The current study provides empirical evidence that sequential patterning of dFNC states can be used to distinguish among people living with PD who present with mild cognitive impairments. Unlike the more common MRI-derived measures of morphology, static functional connectivity, or mean state dwell times, the current analyses revealed specific patterns in the temporal sequencing of dFNC state transitions that are the most discriminative of individuals living with PD, including those who present with mild cognitive impairments. If confirmed by additional independent investigations, these sequential patterns may offer a unique set of features that could be evaluated as candidate endophenotypic markers of abnormal neural network engagement among people living with PD. Accordingly, sequential patterns in the temporal ordering of dFNC states offer a unique feature space for future applications of artificial intelligence methods attempting to identify the defining characteristics of progressive impairment in cognitive functioning.

Our findings contribute to the growing body of literature exploring the dynamic aspects of brain network connectivity in neurodegenerative diseases, particularly among individuals with cognitive impairment. For example, our results align with those reported by Chen and colleagues (2023), who found that individuals with subjective cognitive decline demonstrated altered brain network dynamics, characterized by increased occupancy of states with reduced activation in key networks and impaired transitions into states of general network activation. While both studies highlight the importance of dynamic functional connectivity in early cognitive decline, our results specifically reveal a distinct sequential patterning of transitions between connectivity states which distinguished people with PD from HC individuals with up to 80 % accuracy, as well as distinguishing PD-NC individuals from those with PD-MCI with up to 72 % accuracy. This observation suggests that investigating the sequential patterning of dynamic functional connectivity states may offer a more sensitive approach to monitoring disease progression and the emergence of cognitive impairment in PD, compared to static measures of functional connectivity or averaged dwell times across dFNC states.

Cao and colleagues (2023) have also demonstrated that PD patients exhibit increased time spent in a hypo-connectivity state over time, particularly as non-motor symptoms worsen. Our findings also complement these results by showing that, in addition to prolonged time spent in a hypo-connected state, PD-MCI patients may also spend less time in a state characterized by a pattern of anti-correlated activity between the thalamus and sensory networks (somatomotor, visual, and auditory). These findings are further supported by other studies on neurodegenerative diseases, such as the study by Fiorenzato et al. (2019) which demonstrated dynamic connectivity changes associated with dementia in PD, and the study by Díez-Cirarda and colleagues (2018), which reported reduced inter-network connectivity, particularly between somatomotor, cognitive-control, visual, auditory, and subcortical networks among individuals with PD-MCI. While Díez-Cirarda et al. found that individuals with PD-MCI tended to spend *less* time in a hypo-connected state than healthy control participants, their study used only 2 dFNC states, which may not be directly comparable with the three states identified in the current analysis. Together, these studies highlight that disruptions in dynamic functional network connectivity are not only present but could also serve as early markers for cognitive impairments among people living with PD.

Our findings from the application of machine learning classification using the Seq2Pat method of sequential pattern mining identified distinct sequential patterns of state transitions that accurately differentiated HC individuals from those with PD. Notably, patterns that included transitions from the hyper-connected State 2 to the hypo-connected State 1 and then back to State 2 or State 3 were unique to HC participants, while patterns containing transitions from the hypo-connected State 1 to State 2 (or 3) and then back to State 1 were more discriminative among the PD cohorts for classifying whether or not the individuals also displayed mild cognitive impairments. These findings are also consistent with previous literature suggesting that the disruption of normal sequential connectivity patterns is a hallmark of cognitive decline in neurodegenerative diseases (Filippi et al., 2023). These findings could also be interpreted as lending support to the notion that brain states display wave-like properties such as stereotyped temporal ordering of spatial connectivity patterns and propagation capabilities, which could present unique endophenotypic markers of impairment (Foster and Scheinost, 2024).

Several limitations also should be noted. Participants were scanned with eyes closed, increasing the risk of falling asleep, which could affect connectivity measures. The sliding window technique used to capture dFNC introduces variability based on the chosen window size, which can influence results. Previous studies have identified certain constraints (Hindriks et al., 2016), with one notable concern being the choice of the window size (Damaraju et al., 2014). This parameter determines when network interactions are measured across the scan and is user-controlled, potentially introducing variability into the analysis and consequently, the derived findings. In addition, the *k*-means method and its inherent level of randomness is heavily impacted by data quality and consecutive experiments can only produce similar rather than identical findings when replicated. An additional limitation of the current investigation is that we were not able to compare medication “ON” and “OFF” states, which has been reported to have a significant impact on functional network connectivity measures that directly relate to the presentation of cognitive impairments among people with PD (Kinugawa et al., 2024). All of the participants in the current study were ON their usual prescribed medications during fMRI data collection, but specific dosing information was not available for the calculation of Levodopa Equivalent Daily Dose (LEDD). As such, this is another limitation of the current investigation as we were not able to use this as a co-variate for our analyses.

Recent findings by Chen et al. (2024) have also highlighted the potential importance of hippocampal regions in relation to the presentation of cognitive impairments among people with PD. The potential relationship of hippocampal contributions to cognitive impairments in PD is also of interest to us, as we have previously reported that bilateral hippocampal volumes were significantly lower among people with PD who had MoCA scores less than 26, relative to those with PD who scored 26 or higher on the MoCA (Bona et al., 2022). However, none of the independent components that were identified by GIFT in our analyses were specific to hippocampal networks, thus limiting our ability to make inferences about the contribution of these regions in the current investigation. Future studies using a similar approach could therefore benefit from alternative functional parcellation methods that may help to identify the contribution of hippocampal networks to the sequential patterning of emergent dFNC brain states among people with PD.

Our study’s effect size was large enough to detect differences between the three groups in the comparisons of mean dwell times; however, the small sample does limit the generalizability of the differences in the observed correlation matrices, particularly for State 3 within the PD-MCI group, which had the smallest number of participants and the least observed instances of this state. Nonetheless, the results from most of the tested classifiers show moderate to high accuracies and all were above chance level (Table 2), indicating that the sequential patterning of derived brain states warrants further investigation as a potential marker of abnormality in the dynamic temporal organization of neural network activity among people living with PD. We believe that this analysis highlights the potential utility of characterizing the sequential patterning of dynamic brain states and future research endeavors should consider further developing analysis methods akin to Seq2Pat for this purpose.

## 5 Conclusions

Our investigation used resting state fMRI to determine whether the temporal characteristics or sequential patterning of dFNC states could accurately distinguish among people living with PD who had normal cognition, those living with PD who displayed mild cognitive impairments, and healthy older-aged individuals. Significant differences were found in the time spent in various dFNC states, and a machine-learning method of sequential pattern mining yielded reasonably accurate classifications, revealing distinct sequential patterns in the temporal ordering of dFNC states that distinguished the three groups of study participants. These findings underscore the potential of dFNC and sequential pattern mining to further elucidate the suspected role of disruptions in functional coupling across neural networks as potentially important indicators of cognitive decline among people living with PD. Future research should focus on refining these methods and exploring their potential for clinical applications in early diagnosis and monitoring of cognitive impairments in the clinical progression of Parkinson’s disease.

## CRediT authorship contribution statement

**Aaron S. Kemp:** Writing – review & editing, Visualization, Validation, Software, Methodology, Formal analysis, Data curation. **A. Journey Eubank:** Writing – review & editing, Writing – original draft, Visualization, Validation, Software, Methodology, Formal analysis, Data curation. **Yahya Younus:** Software, Resources. **James E. Galvin:** Writing – review & editing, Supervision, Resources, Methodology, Investigation, Funding acquisition, Conceptualization. **Fred W. Prior:** Writing – review & editing, Supervision, Resources. **Linda J. Larson-Prior:** Writing – review & editing, Supervision, Resources, Methodology, Investigation, Funding acquisition, Conceptualization.

## Funding

Financial support for this project was provided by a competitive research grant from the 10.13039/100000864Michael J. Fox Foundation (PI: Galvin), which had no direct involvement in the collection, analysis, or interpretation of the data presented herein.

## Data Availability

The code used to conduct the Dichotomic Pattern Mining and the machine learning classifications is available to download from the following Github repository: https://github.com/kempaaron/seq2pat_notebook. All MRI data utilized in the current dFNC analyses are also available for download in BIDS format at the following OpenNeuro repository: doi:10.18112/openneuro.ds005892.v1.0.0.
